# Modeling Primary Emissions of Chemicals from Liquid Products Applied on Indoor Surfaces

**DOI:** 10.3390/ijerph191610122

**Published:** 2022-08-16

**Authors:** Wenjuan Wei, John C. Little, Mélanie Nicolas, Olivier Ramalho, Corinne Mandin

**Affiliations:** 1 Scientific and Technical Center for Building (CSTB), Health and Comfort Department, French Indoor Air Quality Observatory (OQAI), University of Paris-Est, CEDEX 2, 77447 Marne la Vallée, France; 2 Department of Civil and Environmental Engineering, Virginia Tech, Blacksburg, VA 24060, USA

**Keywords:** volatile organic compounds, consumer exposure, household cleaning products, personal care products, intermittent source

## Abstract

Liquid products applied on material surfaces and human skin, including many household cleaning products and personal care products, can lead to intermittent emissions of chemicals and peak concentrations in indoor air. The existing case-based models do not allow inter-comparison of different use scenarios and emission mechanisms. In this context, the present work developed a mechanistic model based on mass transfer theories, which allowed emissions into the air from the liquid product to be characterized. It also allowed for diffusion into the applied surface during product use and re-emission from the applied surface after the depletion of the liquid product. The model was validated using literature data on chemical emissions following floor cleaning and personal care product use. A sensitivity analysis of the model was then conducted. The percentage of the chemical mass emitted from the liquid to the air varied from 45% (applied on porous material) to 99% (applied on human skin), and the rest was absorbed into the applied material/skin. The peak gas-phase concentration, the time to reach the peak concentration, and the percentage of the liquid-to-air emission depended significantly on the chemical’s octanol/gas and material/gas partition coefficients and the diffusion coefficient of the chemical in the applied material/skin.

## 1. Introduction

Chemicals in indoor environments, including aldehydes, volatile organic compounds (VOCs), and semi-volatile organic compounds (SVOCs), come from various sources. Common indoor sources include (1) porous solid/soft materials, such as building materials, furniture, and children’s toys; (2) applied and sprayed liquids, such as surface cleaning products, air fresheners, body lotion, and household pesticides; and (3) heating and combustion products, such as incense, gas stoves, and candles [1]. According to the emission characteristics, these indoor sources can be classified into (1) continuous regular sources, such as furniture, which have constant source strength; (2) continuous irregular sources, such as wet paints, which have variable source strength during curing; (3) intermittent regular sources, such as gas stoves, which have a regular time-pattern of use; and (4) intermittent irregular sources, such as household cleaning products, which have variable time-pattern of use [2].

Most of the studies in the past decades focused on characterizing chemical emissions from continuous sources. They include many materials that can emit chemicals continuously to the indoor environment, from when the source materials are placed indoors to when they are removed, resulting in long-term emissions. A pioneering mechanistic model was developed in 1994 to predict the diffusion of VOCs contained in carpets through the source material to a ventilated environment [3]. The model has been improved and extended, forming a series of models that address chemical emissions from continuous regular and irregular sources. The improvements include (1) extending the model to address emissions from other materials, such as multi-layer dry porous materials [4,5,6,7] and paint film coating that transforms from wet to dry states [8,9,10,11,12,13]; (2) extending the model to address the emissions of other chemicals, such as formaldehyde [14,15,16] and SVOCs [17,18,19,20,21,22,23]; (3) considering other mechanisms for chemical emission and transport, such as convective mass transfer in the boundary layer adjacent to the source material [24,25], VOC adsorption on sink surfaces [26,27,28,29,30,31], and SVOC partitioning between the different phases [32,33,34,35]; and (4) considering the influence of environmental factors, such as temperature and relative humidity, on the emission rate [36,37,38,39].

Whereas continuous sources lead to long-term emissions, intermittent sources can cause peak chemical concentrations in the environment when they are used, leading to intense short-term occupant exposure [2]. For intermittent sources, fewer emission models have been developed compared to continuous sources. A possible reason is that the emission mechanism of an intermittent source is dependent on the form of the source, e.g., spray, film, and combustion; the use scenario, i.e., the chemical mass of the emission source that is used, the dimension of the emission source, and the emission period; and the associated environment, e.g., office, living room and bathroom [40]. Among indoor intermittent sources, liquid products applied on material surfaces or human skin for cleaning or personal care purposes can be characterized by surface emission [40]. Three types of models have been developed, i.e., models based on convective mass transfer, diffusion, and evaporation. Models based on convective mass transfer aim to characterize the emission of liquid products during product use [41,42]. Models based on diffusion aim to characterize the re-emission of adsorbed products from permeable surfaces after product use [43,44,45]. Models based on evaporation aim to provide a simplified estimation of the average emission rate [46,47,48,49,50]. Despite the different scope of applications, the case-based models do not allow inter-comparison of different use scenarios and emission mechanisms, and there is a lack of a comprehensive understanding of the emission profiles and exposure characteristics for these liquid products.

Since intermittent chemical sources include household cleaning and personal care products that are commonly used in indoor environments and on human skin, chemicals emitted from these products contribute to the aggregated exposure of occupants to indoor pollutants via multiple pathways. Due to the lack of a holistic modeling framework to address the mass transfer of indoor intermittent sources, the objective of the present work was to develop a mechanistic model to characterize different chemical transfer phenomena following the use of liquid products on material surfaces and human skin. The present work focused on primary emissions of the initial product ingredients. The decomposition of some product ingredients may lead to the formation of other VOCs, such as formaldehyde formation from bronopol decomposition [51]. However, the formation of decomposed chemicals is not discussed in the present work. Once the product ingredients are emitted into the air, they can react with oxidants leading to emissions of secondary chemicals [52]. This chemical reaction process can be characterized using indoor chemistry models [53], but the formation of secondary chemicals after emission is not discussed in the present work.

## 2. Materials and Methods

### 2.1. Model Development

**Figure 1:**
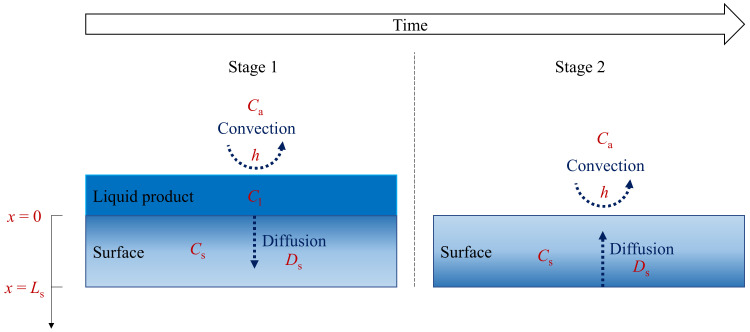
Schematic representation of the model to predict the primary emission of chemicals associated with the use of a liquid product.

The model considers two emission stages for products used on surfaces (Figure 1), i.e., when a product liquid layer exists (stage 1) and after the product liquid layer is depleted (stage 2). During product use (stage 1), a product liquid layer exists on the applied surface, and the model considers three zones, i.e., the product liquid layer as the chemical emission source, the surface material below the liquid where the product ingredients can diffuse through if it is permeable, and the air above the liquid where the chemicals emitted to the air can be characterized by convective mass transfer. After the product application (stage 2), the liquid layer is depleted progressively (the thickness of the liquid layer reduces to zero), and the model considers two zones, i.e., the surface material and the air. If the surface material is permeable and contains adsorbed product ingredients, it re-emits the chemicals to the air as the emission source. If the surface material is impermeable, no more emission source exists in stage 2. Mathematical equations based on diffusion and convective mass transfer theories to characterize the two emission stages are as follows.

The chemical concentration in the surface material or skin is:

(1) $$\frac{\partial C_{s}}{\partial t}=D_{s}\frac{\partial^{2}C_{s}}{\partial x^{2}}$$

where *C*_s_ is the chemical concentration in the material (µg/m^3^), *t* is time (s), *D*_s_ is the chemical diffusion coefficient in the material (m^2^/s), and *x* is the distance below the surface of the material (m). The surface material or skin is considered to be clean before product use, thus, the initial condition is:

(2) $$C_{s}=0,\;\mathrm{if}\;t=0$$

The current model does not consider chemical degradation in the human body if the product is applied to the skin, thus the boundary conditions are:

(3) $$C_{s}=\frac{C_{l}}{K_{\mathrm{ls}}},\;\mathrm{if}\;x=0,\;\mathrm{for}\;\mathrm{stage}\;1$$

(4) $$D_{s}\frac{\partial C_{s}}{\partial x}=h\left(\frac{C_{s}}{K_{\mathrm{sa}}}-C_{a}\right),\;\mathrm{if}\;x=0,\;\mathrm{for}\;\mathrm{stage}\;2$$

(5) $$\frac{\partial C_{s}}{\partial x}=0,\;\mathrm{if}\;x=L_{s}$$

where *C*_l_ is the concentration of the target chemical in the product liquid (µg/m^3^), *L*_s_ is the thickness of the material (m), *K*_ls_ is the liquid/material partition coefficient (-), *K*_sa_ is the material/air partition coefficient (-), and *h* is the convective mass transfer coefficient in the air (m/s).

Due to the diversity of product formulae that are often not accessible, the model cannot consider the emission rate for each product ingredient. Therefore, the mass percentage of the target chemical in the product liquid is considered to be constant, so that the concentration of the target chemical (*C*_l_) in the liquid is constant before the liquid is depleted. This is a mathematical simplification of the multi-ingredient emission process, nevertheless, complete knowledge of the product formulae should be acquired to conduct more complex modeling of multi-ingredient emissions. The chemical mass in the liquid is:

(6) $$m_{l}=m_{0}-\int_{0}^{t}\left[hA\left(\frac{C_{l}}{K_{\mathrm{oa}}}-C_{a}\right)+\int_{0}^{L_{s}}A\frac{\partial C_{s}}{\partial t}dx\right]dt$$

where *m*_l_ is the chemical mass in the liquid (µg), *m*_0_ is the initial chemical mass in the liquid (µg), *K*_oa_ is chemical octanol/air partition coefficient (-), *A* is the surface area (m^2^), *C*_a_ is the chemical concentration in the air (µg/m^3^). The chemical concentration in the air is:

(7) $$\frac{dC_{a}}{dt}=Q\left(C_{\mathrm{out}}-C_{a}\right)+hA\left(\frac{C_{l}}{K_{\mathrm{oa}}}-C_{a}\right),\;\mathrm{for}\;\mathrm{stage}\;1$$

(8) $$V\frac{dC_{a}}{dt}=Q\left(C_{\mathrm{out}}-C_{a}\right)+hA\left(\frac{C_{s}}{K_{\mathrm{sa}}}-C_{a}\right),\;\mathrm{for}\;\mathrm{stage}\;2$$

where *V* is the air volume (m^3^), *Q* is the air flow rate in the indoor environment (m^3^/s), *C*_out_ is the chemical concentration in the outdoor air that enters the environment (µg/m^3^), and *K*_sa_ is the material/air partition coefficient (-). The indoor air is considered to be clean before product use, thus, the initial condition is:

(9) $$C_{a}=0,\;\mathrm{if}\;t=0$$

The model implies the following assumptions for its current development: (1) the surface material or skin and the indoor air are considered to be clean before product use, thus the current model should be used independently and is not to be coupled with other emission models; (2) chemical diffusion into and out of the surface material or skin is considered a one-dimensional diffusion driven by the concentration gradient of the chemical; (3) chemical degradation in the human body is neglected if the product is applied to the skin, thus it may underestimate the chemical mass that enters the skin in stage 1 and overestimate the chemical mass that exists the skin in stage 2; and (4) the mass percentage of the target chemical in the product liquid is constant during the liquid emission process, thus the prediction error in the emission profile depends on the product formulae and may be significant if the product contains ingredients that have diverse volatilities. The four assumptions could be relaxed, but this would lead to models that are more complex mathematically, requiring detailed information on product formulation and human physiological parameters.

Numerical solutions of the differential equations were obtained by the finite-difference method applying the central difference approximation. After conducting simulations with different numbers of layers, the material or skin was divided into 10 layers along its thickness, thus the spacing is 1/10 of the thickness because more simulation layers did not improve the results. The time step for the calculation varied with materials and chemicals and was changed for each calculation to ensure that the results would converge.

The model requires 13 input parameters (Table 1). Nine parameters are associated with indoor/outdoor environments, i.e., the chemical’s initial concentration in the material/skin, chemical concentration in the liquid, material/skin thickness and area, liquid mass applied, convective mass transfer coefficient, indoor volume, indoor air flow rate, and the chemical’s initial concentrations in indoor and outdoor air. Only four parameters are associated with chemical and material properties, i.e., the chemical’s diffusion coefficient in the material/skin, the chemical’s octanol/gas partition coefficient, the chemical’s material/gas partition coefficient, and the chemical’s liquid/material partition coefficient, which needs to be obtained from theoretical or experimental studies.

### 2.2. Model Validation Using Measured Data

The model was validated using experimental data from two studies obtained from the literature. The first study measured acetic acid (a common ingredient in cleaning products) concentration profiles in indoor air in a house following the use of an all-purpose cleaner on hardwood flooring [46]. The study conducted measurements for only 6 min following product use, thus the results were used to validate only stage 1 of the present model. The second study measured decamethylcyclopentasiloxane (D5, a common ingredient in cosmetic products) concentration profiles in indoor air in a university classroom following the use of personal care products on human skin [45]. The study determined the initial D5 concentration in human skin after the depletion of the product liquid, thus the results were used to validate only stage 2 of the present model. The two stages of the model were validated using experimental data from two different studies because no study was found to measure both liquid emission and surface re-emission of the product ingredients. The input data for the model prediction were obtained mainly from the two studies and are shown in Table 1. The chemical’s octanol/gas partition coefficient was obtained using the EPI Suite calculator developed by the U.S. Environmental Protection Agency [54].

**Table 1:** Input data for modeling indoor concentrations associated with product use.

Parameter	Acetic Acid Emission from Floor Cleaning [46]	Decamethylcyclopentasiloxane (D5) Emission from Human Skin [45]
Diffusion coefficient in the material/skin (m^2^/s)	5.05 × 10^−10^ [55]	1.46 × 10^−16^ [45]
Initial chemical concentration in the material/skin (µg/m^3^)	0 (Assumed)	8.80 × 10^10^ [45]
Octanol/gas partition coefficient, *K*_oa_ (-)	1.66 × 10^5^ (Calculated using EPI Suite)	8.57 × 10^6^ (Calculated using EPI Suite)
Material/gas partition coefficient, *K*_sa_ (-)	6.27 × 10^2^ [55]	3.27 × 10^4^ [45]
Liquid/material partition coefficient, *K*_ls_ (-)	2.65 × 10^2^ (Estimated as *K*_oa_/*K*_sa_)	2.65 × 10^2^ (Estimated as *K*_oa_/*K*_sa_)
Chemical concentration in the liquid (µg/m^3^)	4.2 × 10^10^ [46]	Not needed for the model in stage 2
Material/skin thickness	1.9 cm [55]	1 µm [45]
Material/skin area (m^2^)	5.6 × 10^−1^ [46]	2.28 (24 students) [45]
Liquid mass applied (µg)	2.52 × 10^6^ [46]	Not needed for the model in stage 2
Convective mass transfer coefficient (m/s)	9 × 10^−4^ [45]	9 × 10^−4^ [45]
Indoor volume (m^3^)	2.45 × 10^1^ [46]	670 [45]
Air flow rate (m^3^/s)	8.33 × 10^−3^ [46]	0.93 [45]
Initial chemical concentration in indoor air (µg/m^3^)	0 [46]	0 [45]

## 3. Results and Discussion

### 3.1. Predicted Indoor Concentrations Following Product Use

**Figure 2:**
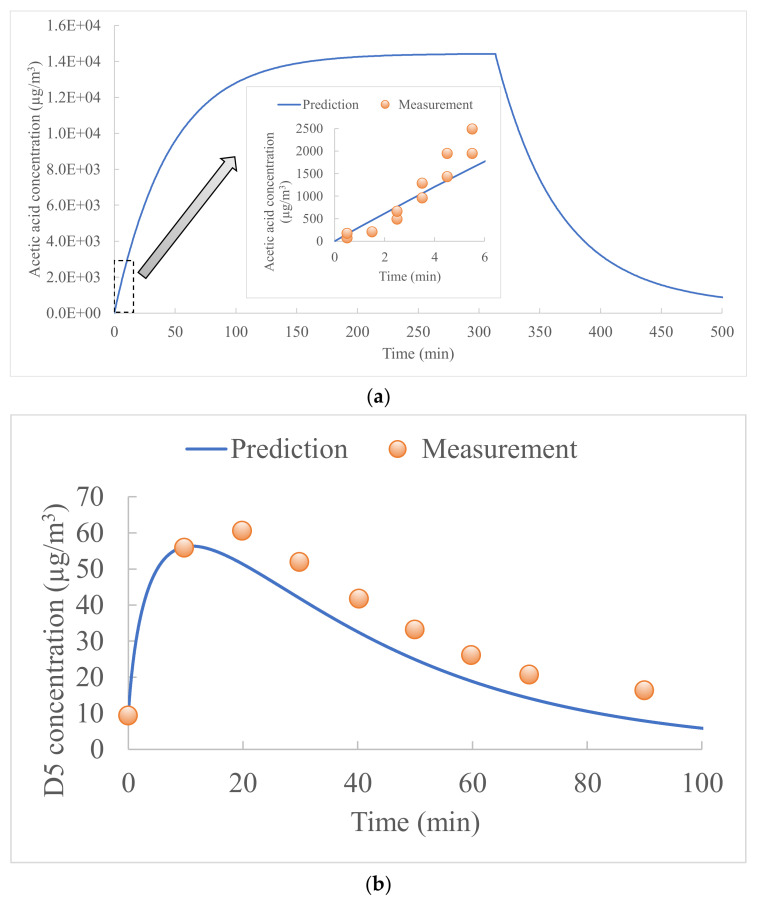
Indoor gas-phase concentrations of (**a**) acetic acid and (**b**) decamethylcyclopentasiloxane (D5).

Following the use of all-purpose cleaner on hardwood flooring [46] and personal care products [45] respectively in two indoor environments, the gas-phase concentrations of acetic acid and D5 were predicted using the present model and compared to the measured data in the literature. Figure 2a shows the predicted gas-phase concentration profile of acetic acid in indoor air during the entire emission process. The acetic acid in the air reached the peak gas-phase concentration 5 h after the use of 60 mL of a cleaning product containing 2.52 g of acetic acid on a hardwood floor of 0.56 m^2^ in a room of 24.5 m^3^ with an air change rate of 0.5 h^−1^. During this liquid emission period (stage 1), 90.7% of the initial acetic acid mass was emitted from the liquid to the air. After the liquid depletion, the acetic acid diffused into the wood was re-emitted into the air. The wood re-emission period (stage 2) lasted 5 h, and 9.3% of the initial acetic mass was re-emitted from the floor. The predicted acetic acid concentrations were compared with the measured concentrations for 6 min after the use of the product, and the differences were within a factor of two. A possible explanation of the difference is that the measurement study did not provide data on the diffusion coefficient and partition coefficient of acetic acid in the hardwood floor, as well as the thickness of the floor. These three input parameters were obtained from another emission measurement of acetic acid from wood furniture [55], and the differences in the material properties may lead to bias in the prediction.

Figure 2b shows the comparison of the predicted and measured gas-phase concentrations of D5 in the air of a classroom of 670 m^3^ with an air change rate of 5 h^−1^. Since the D5 was re-emitted from human skin according to the measurement study, the prediction did not consider liquid emission. The D5 in the air reached the peak gas-phase concentration 10 min after emission according to the predictions, and 20 min according to the measurements. Then the D5 concentrations decreased for 1 h to reach the background concentration in the classroom. The predicted and measured concentrations were well correlated, and the differences were less than 30% for each sampling point.

Measurements of the two above-mentioned studies in the literature allowed the present model to be partially validated. Due to the lack of existing experimental data measuring both liquid-to-air and liquid-to-surface mass transfers in stage 1 and surface-to-air emission in stage 2, full validation of the present model can be challenging. The lack of experimental data and the need to design a complete experimental study in the future highlights the necessity of conducting a sensitivity analysis at the current stage to characterize the influence of the chemical and surface material properties on the emission profile.

### 3.2. Sensitivity Analysis of Parameters Influencing the Emission

The present model requires 13 input parameters, as shown in Table 1. Depending on whether the liquid product was used on a wood floor or human skin, significant differences were observed in five parameters, i.e., the chemical’s diffusion coefficient in the material/skin, the chemical’s octanol/gas, and material/gas partition coefficients, the material/skin thickness, and the indoor air flow rate (Table 1). A sensitivity analysis of these 5 parameters was conducted to study their influences on the emission profile.

#### 3.2.1. Chemical Diffusion Coefficient in the Material/Skin

**Figure 3:**
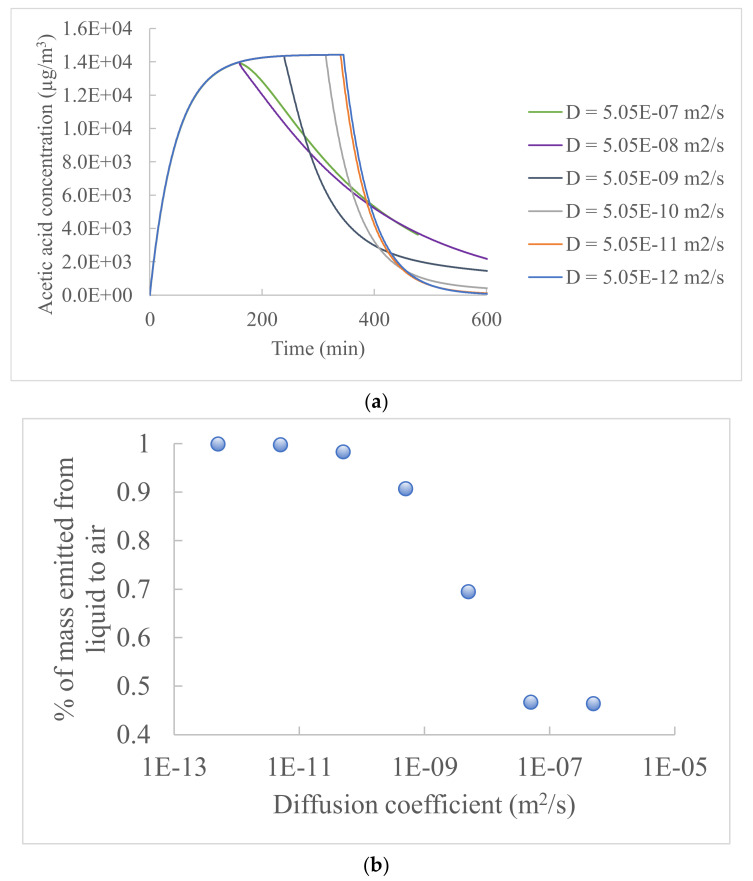
Influence of diffusion coefficient (*D*) on the indoor gas-phase concentration of acetic acid: (**a**) concentration profile and (**b**) fraction of mass emitted from the product liquid to air.

Figure 3 shows the indoor gas-phase concentration profiles of acetic acid, for which the chemical’s diffusion coefficient in the material/skin varied between 5.05 × 10^−7^ and 5.05 × 10^−16^ m^2^/s (the orders of magnitude can cover indoor materials and skin), and the other input parameters had the same values as in the study of acetic acid emission from floor cleaning (Table 1). The sensitivity analysis of the diffusion coefficient aimed to characterize the influence of the material’s permeability on the emission profile. During the liquid emission period (stage 1), the concentration profiles were identical regardless of the material’s diffusion coefficient until the product liquid was depleted and the peak gas-phase concentration was reached. The time to reach the peak concentration depended on the diffusion coefficient and varied between 159 and 346 min (Figure 3a). The increased diffusion coefficient resulted in a higher rate of chemical diffusion through the material, thus faster depletion of the chemical in the liquid product. The peak concentration value in the gas phase was hardly influenced by the diffusion coefficient since the chemical emission from the liquid to the air during stage 1 was due to airflow on the surface of the product liquid. The percentage of the total chemical mass emitted from the product liquid to the air was also dependent on the diffusion coefficient and varied between 46.4% and 99.9% (Figure 3b). The rest of the chemical mass was absorbed into the material/skin and was re-emitted from the material/skin to the air during stage 2. When the diffusion coefficient was less than 10^−11^ m^2^/s, the percentage of the liquid-to-air emission reached 99.7% and was hardly influenced by the decreased diffusion coefficient. Since many building materials are porous and have diffusion coefficients higher than 10^−11^ m^2^/s, a non-neglectable proportion of the chemical applied on indoor material surfaces can be absorbed into the materials and re-emitted to the air after the liquid depletion. However, when the product liquid is applied to human skin, most of the chemical is emitted into the air from the liquid, as human skin has low permeability and low diffusion coefficients between 10^−14^ and 10^−16^ m^2^/s [42]. During the material re-emission period (stage 2), materials with high diffusion coefficients contained more absorbed chemical mass compared to materials with low diffusion coefficients, leading to a higher material re-emission and a slower decrease of the indoor gas-phase concentrations.

#### 3.2.2. Chemical’s Octanol/Gas and Material/Gas Partition Coefficients

**Figure 4:**
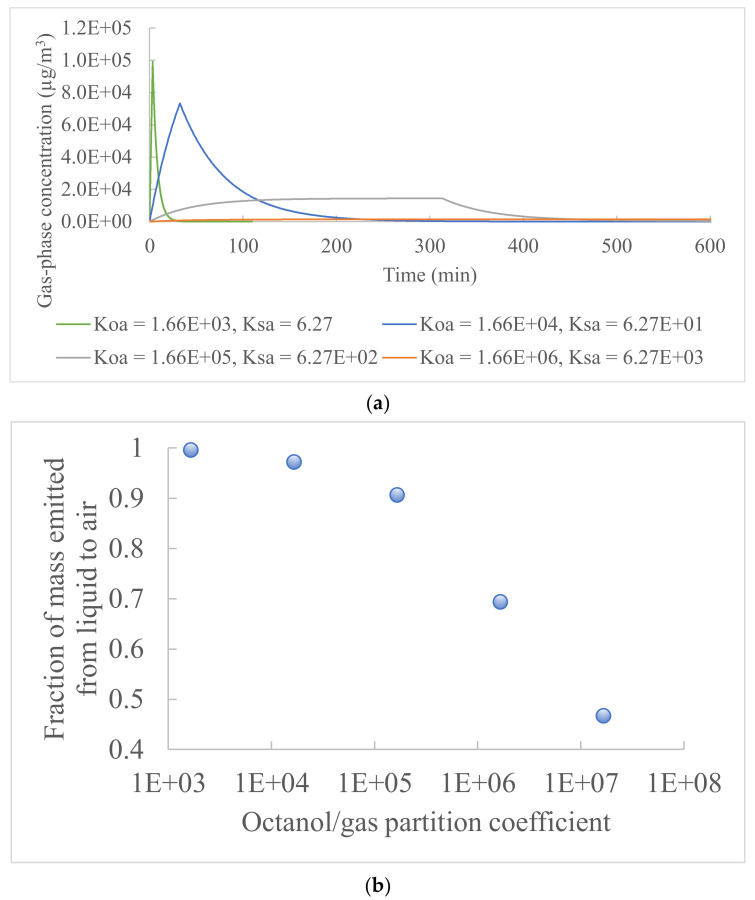
Influence of octanol/gas and material/gas partition coefficients (*K*_oa_ and *K*_sa_) on the chemical indoor gas-phase concentration: (**a**) concentration profile and (**b**) fraction of mass emitted from the product liquid to air.

Figure 4 shows the indoor chemical gas-phase concentration profiles, for which the chemical’s octanol/gas partition coefficient varied between 1.66 × 10^3^ and 1.66 × 10^7^, and the material/gas partition coefficient varied between 6.27 and 6.27 × 10^4^. The two partition coefficients had large ranges to cover volatile chemicals and were varied together to maintain a constant ratio so that the liquid/material partition coefficient remained unchanged for the analysis. The other input parameters had the same values as in the study of acetic acid emission from floor cleaning (Table 1). The two partition coefficients can significantly affect the peak gas-phase concentrations, the time to reach the peak concentration, and the percentage of the chemical mass emitted from the product liquid to the air (Figure 4). This is because chemicals with low partition coefficients are more volatile than those with high partition coefficients and tend to be emitted from the liquid to the air. During the liquid emission period (stage 1), decreased octanol/gas and material/gas partition coefficients led to a shorter time for the liquid to be depleted (from 4.5 days to 3 min) and higher chemical peak concentrations in the gas phase (from 1.44 × 10^2^ to 9.92 × 10^4^ µg/m^3^) (Figure 4a). The percentage of the chemical mass emitted from the product liquid to the air decreased with higher octanol/gas and material/gas partition coefficients and varied between 46.7% and 99.6% (Figure 4b). During the material re-emission period (stage 2), materials with higher material/gas partition coefficients contained more absorbed chemical mass compared to materials with low material/gas partition coefficients, leading to a higher material re-emission and a slower decrease of the indoor gas-phase concentrations.

#### 3.2.3. Material/Skin Thickness

The material/skin thickness varied between 1.90 × 10^3^ and 1.90 × 10^−1^ mm for the sensitivity analysis, and the other input parameters had the same values as in the study of acetic acid emission from floor cleaning (Table 1). Changes in the material/skin thickness by 5 orders of magnitude led to less than 1% difference in the peak gas-phase concentrations, less than 30 min difference in the time to reach the peak concentrations, and less than 10% difference in the percentage of the chemical mass emitted from the product liquid to the air. Therefore, the material/skin thickness had much less influence on the liquid-to-air emission and liquid-to-material diffusion processes, compared to the chemical diffusion and partition coefficients.

#### 3.2.4. Indoor Air Flow Rate

**Figure 5:**
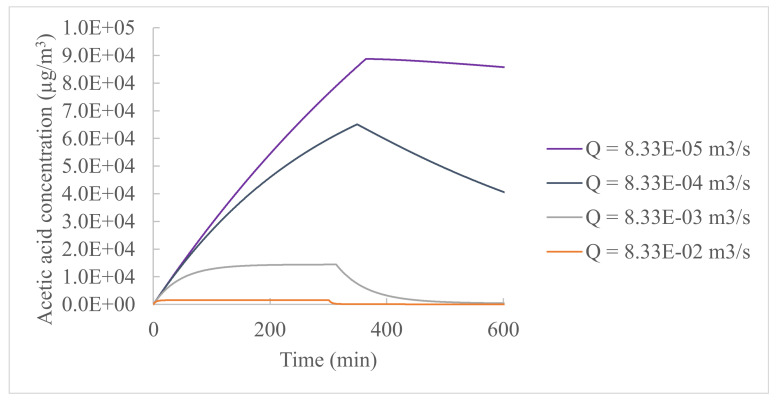
Influence of the airflow rate (Q) on the chemical indoor gas-phase concentration.

Figure 5 shows the indoor gas-phase concentration profiles of acetic acid, for which the indoor air flow rate varied between 8.33 × 10^−5^ and 8.33 × 10^−1^ m^3^/s (0.0122–122 air change per hour), and the other input parameters had the same values as in Table 1. The indoor air flow rate can significantly affect the peak gas-phase concentration value by orders of magnitude and change the time to reach the peak concentrations by hundreds of minutes. Changes in the airflow rate by 5 orders of magnitude led to less than a 2% difference in the percentage of the total chemical mass emitted from the product liquid to the air.

## 4. Conclusions

A mechanistic model was developed to characterize the chemical emission profiles following the use of liquid products on indoor surfaces and human skin. The model characterizes the liquid-to-air emission and the liquid-to-surface diffusion during the product use, as well as the surface-to-air emission after the depletion of the product liquid. The multiple chemical emission phenomena considered in the model allow it to address occupants’ exposure via multiple pathways for indoor intermittent sources applied on surfaces, including household cleaning and personal care products. The simulated indoor gas-phase chemical concentrations agreed with the measured indoor concentrations of acetic acid following the use of a cleaning product on hardwood flooring, as well as the measured indoor concentrations of D5 due to the use of personal care products on human skin.

Following the product use, the chemical’s emission profile depended significantly on the chemical’s diffusion coefficient in the material/skin, the chemical’s octanol/gas and material/gas partition coefficients, and the indoor air flow rate. The partition coefficients affected the peak gas-phase concentration by orders of magnitude and the time to reach the peak concentration by hours.

The percentage of the chemical mass emitted from the product liquid to the air increased with decreasing octanol/gas partition coefficients and was higher than 90% for volatile chemicals whose octanol/gas partition coefficients were lower than 10^−5^. The increased diffusion coefficient reduced the time to reach the peak gas-phase concentration by hundreds of minutes but had a minor influence on the peak concentration value. The percentage of the chemical mass emitted from the product liquid to the air increased with decreasing diffusion coefficient and was higher than 90% if the product liquid was applied on materials whose diffusion coefficients were lower than 10^−10^ m^2^/s, e.g., human skin. The gas-phase concentrations also decreased with increasing air flow rate by orders of magnitude. The results suggest that when volatile chemicals are applied on surfaces with low permeability, most of the chemicals may be emitted from the product liquid to the air as a primary emission associated with the product use. When chemicals with low volatility are applied on surfaces with high permeability, the material absorption can be significant, and the emission can last long after the depletion of the product liquid. The results have highlighted that the use of cleaning and personal care products can result in exposure via inhalation and dermal contact, and the percentage of the contribution of the two exposure pathways is dependent on the chemical and skin properties.
